# Ontogeny of Toll-Like and NOD-Like Receptor-Mediated Innate Immune Responses in Papua New Guinean Infants

**DOI:** 10.1371/journal.pone.0036793

**Published:** 2012-05-23

**Authors:** Joanne G. Lisciandro, Susan L. Prescott, Marie G. Nadal-Sims, Catherine J. Devitt, William Pomat, Peter M. Siba, Meri C. Tulic, Patrick G. Holt, Deborah Strickland, Anita H. J. van den Biggelaar

**Affiliations:** 1 Telethon Institute for Child Health Research, Centre for Child Health Research, University of Western Australia, Perth, Australia; 2 School of Paediatrics and Child Health, University of Western Australia, Perth, Australia; 3 Papua New Guinea Institute of Medical Research, Goroka, Papua New Guinea; University of Massachusetts Medical School, United States of America

## Abstract

Studies addressing the ontogeny of the innate immune system in early life have reported mainly on Toll-like receptor (TLR) responses in infants living in high-income countries, with little or even no information on other pattern recognition receptors or on early life innate immune responses in children living under very different environmental conditions in less-developed parts of the world. In this study, we describe whole blood innate immune responses to both Toll-like and nucleotide-binding oligomerization domain (NOD)-like receptor agonists including the widely used vaccine adjuvant ‘alum’ in a group of Papua New Guinean infants aged 1–3 (n = 18), 4–6 (n = 18), 7–12 (n = 21) and 13–18 (n = 10) months old. Depending on the ligands and cytokines studied, different age-related patterns were found: alum-induced IL-1β and CXCL8 responses were found to significantly decline with increasing age; inflammatory (IL-6, IL-1β, IFN-γ) responses to TLR2 and TLR3 agonists increased; and IL-10 responses remained constant or increased during infancy, while TNF-α responses either declined or remained the same. We report for the first time that whole blood innate immune responses to the vaccine adjuvant alum decrease with age in infancy; a finding that may imply that the adjuvant effect of alum in pediatric vaccines could be age-related. Our findings further suggest that patterns of innate immune development may vary between geographically diverse populations, which in line with the ‘hygiene hypothesis’ particularly involves persistence of innate IL-10 responses in populations experiencing higher infectious pressure.

## Introduction

The human immune system undergoes developmental changes in postnatal life with some responses reaching adult-like profiles at early age and others continuing to develop into late childhood. This involves maturation of both the adaptive immune system that is largely naïve at birth and acquires memory to an increasing variety of environmental exposures over time; and the innate immune system, for which there is growing evidence that functioning at birth is distinct from that of an adult. Several studies have characterized innate immune responses to toll-like receptor (TLR)-ligands in human neonates and adults; reporting that the production of pro-inflammatory cytokines including IFN-γ, IL-12 and in some studies also TNF-α, is generally impaired in neonates, while the production of IL-1β, IL-6, IL-10 and IL-23 is equal to or even higher than that produced by adults [1]–[4]. This profile of innate cytokine response in newborns favours T helper (Th) 2 rather than protective Th1 responses [5]–[7], which likely accounts for the high susceptibility of newborns to infectious agents [1].

Most studies on the ontogeny of the human innate immune system have been performed in high-income countries. Little is known about whether these developmental patterns also apply to children born in low-income countries where the burden of infectious diseases is considerably higher, particularly in early life [8], [9]. One recent study reporting on TLR-mediated immune responses in the first 12 months of life in infants in the Gambia, Africa [10] found some patterns of immune maturation to be different from what has been described for infants in the western world [11]–[13]: instead of declining innate IL-10 and IL-6, and gradually increasing TNF-α and IFN-γ responses over the first year of life, responses in Gambian infants remained stable between 1 and 12 month of age after TNF-α and IFN-γ production had increased during the first month of life. Moreover, there is some evidence that environmental and lifestyle factors influence neonatal immune function (reviewed in [14], [15]), as well as that infant innate immune responses to Bacillus Calmette-Guérin (BCG) vaccination varies between populations [16]–[18]. Altogether these studies lend support to the hypothesis that innate immune maturation is not uniform across populations of high-income versus low-income settings.

There are currently no studies reporting on innate immune responses to pattern recognition receptors (PRRs) other than TLRs in young infants [19]. Nucleotide-binding oligomerization domain (NOD)-like receptors (NLRs) are PRRs that include both NOD and NALP (NACHT-leucine rich repeat and pyrin-domain containing proteins) receptors that detect microbial products and endogenous danger signals [20]–[22]. NALPs form caspase-1-activating multiprotein complexes (‘inflammasome’) which process pro-inflammatory cytokines such as IL-1β and IL-18 to their mature active form, and were described to mediate the immuno-stimulatory properties of aluminium (‘alum’) adjuvants [23]–[25]. Interestingly, despite the fact that alum is widely used in pediatric vaccines, there is currently no data available on alum-induced responses in infancy.

Studying early immune development in low-income settings may provide significant information on: (a) the pathways responsible for the high susceptibility of newborns and infants to infectious diseases; (b) responsiveness to vaccine adjuvants such as alum and other candidate TLR and NLR agonists [26]; and (c) the trajectory of early immune development and how this relates to susceptibility to non-communicable inflammatory diseases such as allergy in later life [27]–[30]. In this study, we aimed to further explore the ontogeny of TLR and NLR-mediated responses during infancy in a low-income country by describin*g* whole blood cytokine responses in Papua New Guinean (PNG) infants aged 1–18 months old.

## Methods

### Ethics Statement

Written informed consent was obtained from all parents of children participating in this study. Ethical approvals for this study were obtained from the PNG Institute of Medical Research Institutional Review Board (0714), the PNG Medical Research Advisory Council (MRAC No 07/10 and No 09.18) and the Ethics Committee of the Princess Margaret Hospital for Children, Perth, Western Australia (1431/EP).

**Table 1:** Infant characteristics.

	Age group			
	1–3 months	4–6 months	7–12 months	13–18 months
Weight, kg (mean; SD)	6.1 (0.8)	7.2 (1.0)	8.7 (0.9)	9.6 (0.9)
Length, cm (mean; SD)	61.8 (3.3)	64.7 (3.5)	70.2 (4.2)	77.4 (4.0)
Body temperature, °C (mean; SD)	36.4 (0.3)	35.7 (2.4)	36.2 (0.8)	36.0 (0.7)
Still breastfed	16/17 (94%)	17/17 (100%)	13/15 (87%)	6/6 (100%)
Solid food introduced	9/17 (53%)	13/17 (76%)	15/15 (100%)	6/6 (100%)
Hospitalized at least once	3/15 (20%)	0/16 (0%)	3/14 (21%)	1/6 (17%)

### Study Design

This was a cross-sectional follow-up study of children participating in an earlier neonatal immunology study (n = 119), performed between 2008 and 2009 in Goroka, PNG. In this area, respiratory pathogens as well as intestinal helminthes and protozoa are highly endemic: however, malaria is not prevalent due to its location at high altitude (1500–1900 m above sea level). Infants were followed-up in October-November 2009, when they were between 1 and 18 months old. After parental consent was obtained, follow-up visits to the PNG Institute of Medical Research (IMR) clinic were arranged. A total of 83 children were re-located. During this visit, infants were physically examined by a study nurse, information on the child’s vaccination history, medications and illnesses was collected, and a venous blood sample was obtained where possible. Children were excluded from the follow-up study if they had been diagnosed with a congenital illness, were known to be HIV positive, had fever-related illness at the time of follow-up, were no longer living in the study area or where parents did not re-consent. Venous blood samples were obtained from a total of 67 children (male n = 31, female n = 27, data missing for 9 children) varying in age between 1 and 18 months old: aged 1–3 (n = 18), 4–6 (n = 18), 7–12 (n = 21) and 13–18 (n = 10) months.

**Figure 1:**
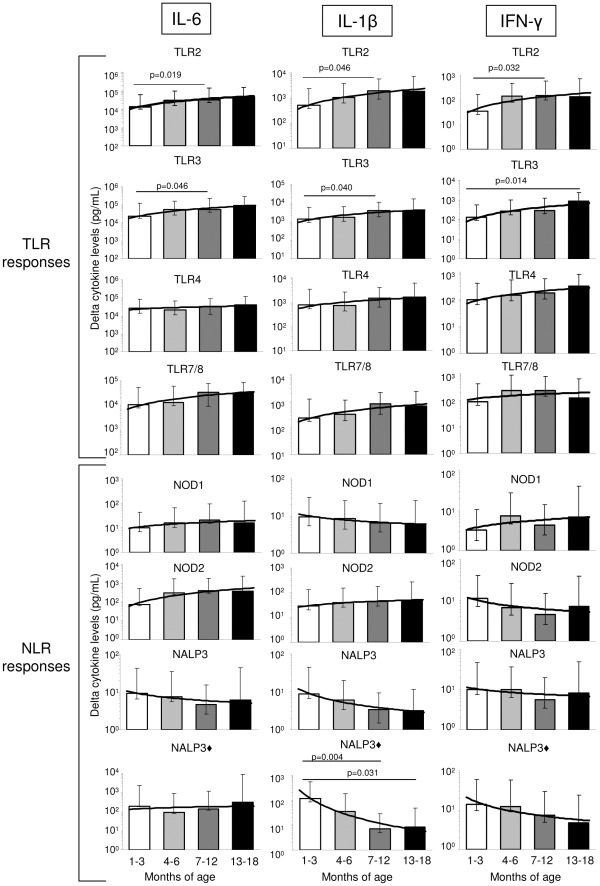
Maturation of innate immune function in PNG infants. Whole blood samples from PNG infants aged 1–3 months (n = 18, *white bars*), 4–6 months (n = 18, *light grey bars*), 7–12 months (n = 21, *dark grey bars*) or 13–18 months (n = 10, *black bars*) were stimulated with TLR (LTA; PolyIC; LPS; Gardiquimod) and NLR (iE-DAP; MDP) ligands, and Alum alone or with LPS co-stimulation (denoted by ♦). Presented are the geometric means and 95% confidence intervals (pg/mL) for each age group for background-adjusted cytokine responses. Significance level is indicated where p<0.05.

**Table 2:** Correlations between innate inflammatory cytokine responses and age.

	IL-6		IL-1β		IFN-γ	
	Rho(slope)	p-value	Rho(slope)	p-value	Rho(slope)	p-value
TLR2	***0.271***	***0.027***	0.237	0.054	***0.248***	***0.043***
TLR3	***0.255***	***0.039***	***0.257***	***0.035***	***0.273***	***0.025***
TLR4	0.122	0.325	0.135	0.277	0.161	0.192
TLR7/8	0.173	0.164	0.176	0.161	0.049	0.698
NOD1	0.088	0.477	−0.118	0.340	0.082	0.507
NOD2	0.159	0.199	0.086	0.488	−0.139	0.261
NALP3	−0.099	0.429	−0.151	0.225	−0.071	0.572
NALP3♦	−0.014	0.913	−***0.368***	***0.002***	−0.158	0.201

Spearman rho correlation coefficients for the slope/trajectory of log-transformed inflammatory cytokine responses across ordered age groups: 1–3, 4–6, 7–12 and 13–18 months were determined. Significance level is indicated; bold-faced and italicized text highlight correlations with p<0.05.

♦ denotes LPS co-stimulation used.

Children were vaccinated according the recommended national immunization schedule, which changed during the period that this birth cohort was born: whereas immunization with BCG and Hepatitis B vaccine (HBV) remained recommended at birth, a neonatal dose of oral polio vaccine was omitted from the later schedule; and immunization with a pentavalent diphtheria, tetanus, whole cell pertussis, *Haemophilus influenzae* type B and hepatitis B combination vaccine (DTwP/HepB/Hib) at 1, 2, and 3 months replaced recommended immunization with separate DTwP and Hib vaccines at 1, 2, and 3 months of age and HBV at 1 and 3 months of age. Measles immunization remained recommended at 6 and 9 months of age.

**Table 3:** Innate IL-10 responses in relation to increasing age in infancy.

IL-10 (pg/mL)	1–3 months	4–6 months	7–12 months	13–18 months	Rho (slope)
TLR2	468 (148–1476)	1168 (686–1990)	1129 (735–1736)	1222 (862–1734)	0.124
TLR3	1554 (869–2779)	2014 (1041–3897)	1856 (812–4240)	3110 (2256–4288)*	0.230
TLR4	581 (208–1624)	691 (327–1461)	587 (386–894)	738 (394–1384)	−0.092
TLR7/8	553 (158–1934)	755 (306–1863)	1146 (810–1622)	1530 (861–2716)	0.032
NOD1	2 (1–3)	2 (1–3)	4 (2–9)	2 (1–5)	0.279‡
NOD2	9 (4–23)	13 (6–30)	10 (4–23)	10 (2–44)	−0.008
NALP3	2 (1–3)	2 (1–2)	2 (1–2)	2 (2–2)	−0.085
NALP3 ♦	36 (9–136)	23 (5–97)	49 (16–151)	35 (5–260)	0.011

Presented are the geometric means and 95% confidence intervals (pg/mL) for background-adjusted IL-10 responses. Mann-Whitney U tests for significant differences in log-transformed IL-10 levels compared to the “1–3 months” age group; and Spearman rho tests for significant correlations between log-transformed IL-10 levels and ordered age groups were conducted. Significance level is indicated where p<0.05 only (* p = 0.046; **‡** p = 0.022).

♦ denotes LPS co-stimulation used.

**Table 4:** Innate TNF-α responses in relation to increasing age in infancy.

TNF-α (pg/mL)	1–3 months	4–6 months	7–12 months	13–18 months	Rho (slope)
TLR2	102 (33–317)	135 (62–294)	81 (34–193)	104 (31–346)	−0.081
TLR3	447 (181–1107)	540 (213–1370)	494 (227–1075)	476 (127–1782)	−0.008
TLR4	224 (106–473)	100 (34–294)	59 (23–147)*	115 (41–321)	−0.207
TLR7/8	182 (76–437)	110 (42–288)	99 (39–249)	47 (9–260)	−0.178
NOD1	2 (1–2)	2 (2–4)	3 (2–4)	3 (2–5)	0.148
NOD2	3 (2–7)	8 (4–17)	5 (2–10)	8 (2–25)	0.140
NALP3	3 (2–5)	2 (1–3)	3 (2–4)	2 (1–5)	0.012
NALP3 ♦	17 (4–76)	11 (3–39)	11 (4–32)	8 (2–34)	−0.054

Presented are the geometric means and 95% confidence intervals (pg/mL) for background-adjusted TNF-α responses. Mann-Whitney U tests for significant differences in log-transformed TNF-α levels compared to the “1–3 months” age group; and Spearman rho tests for significant correlations between log-transformed TNF-α levels and ordered age groups were conducted. Significance level is indicated where p<0.05 only (* p = 0.049).

♦ denotes LPS co-stimulation used.

### Whole Blood Cultures

Infant venous blood samples (2–5 ml) were collected into sterile tubes containing 100 IU preservative-free heparin. Within 2 hrs from collection, an aliquot of blood (500–750 µl) was diluted 5x in RPMI and plated into 96-well round bottom plates (200 µl/well).

**Figure 2:**
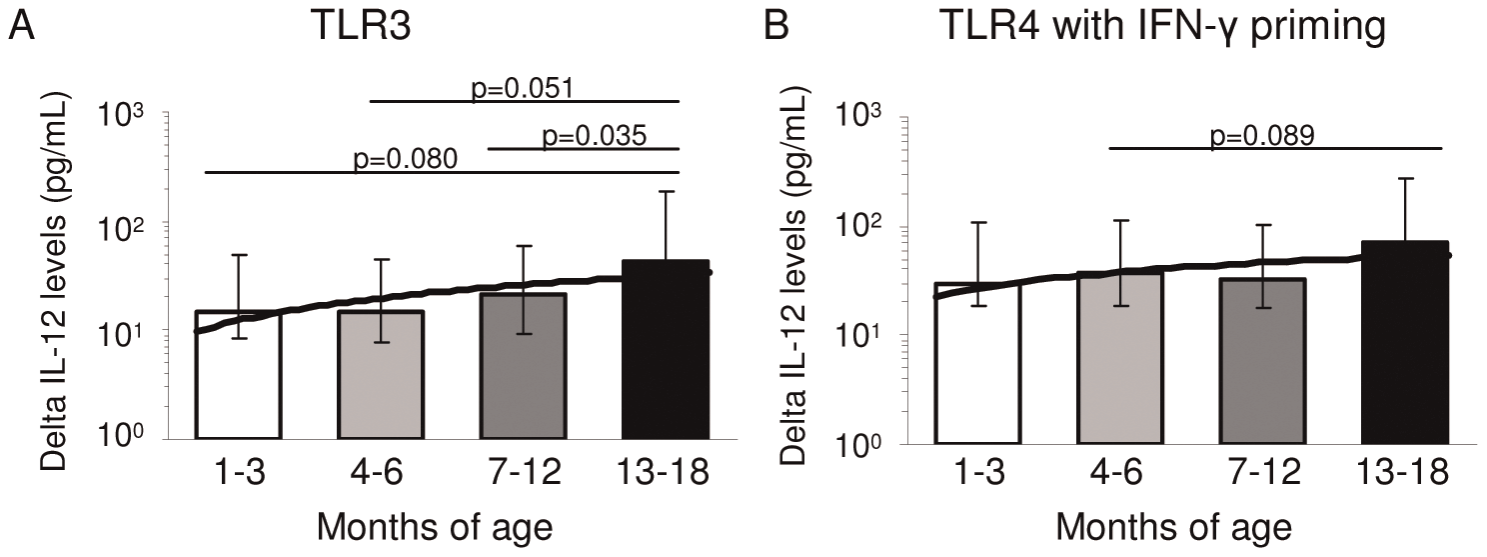
Maturation of innate IL-12 producing capacity during infancy. Whole blood samples from PNG infants aged 1–3 months (n = 18, *white bars*), 4–6 months (n = 18, *light grey bars*), 7–12 months (n  = 21, *dark grey bars*) or 13–18 months (n = 10, *black bars*) were stimulated with (A) polyIC or (B) LPS with IFN-γ priming. Presented are the geometric means and 95% confidence intervals (pg/mL) for each age group for background-adjusted IL-12 responses. Significance level is indicated where p<0.10.

**Figure 3:**
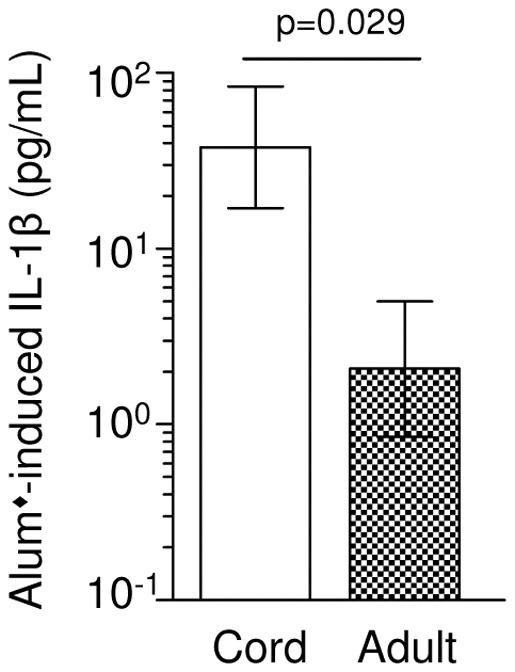
Comparison of PNG cord and adult alum-induced IL-1β production. Cord and peripheral blood mononuclear cells from PNG newborns (n = 47) and PNG adults (n = 5), respectively were stimulated with alum + LPS. Presented are the geometric means and 95% confidence intervals (pg/mL) for each age group for background-adjusted IL-1β responses (i.e. LPS response was subtracted). Significance level is indicated.

For ligands not used previously in any of our studies, cultures were first optimized by comparing innate cytokine responses (IL-6, IL-10, IFN-γ and TNF-α) in cell cultures stimulated with three increasing doses of the ligand during 6, 24 or 48 hours: 24 hours was identified as the single time point giving the highest immune responses for most ligand-cytokine combinations, and concentrations inducing the overall best cytokine response were selected as indicated for further experiments (data not shown).

**Figure 4:**
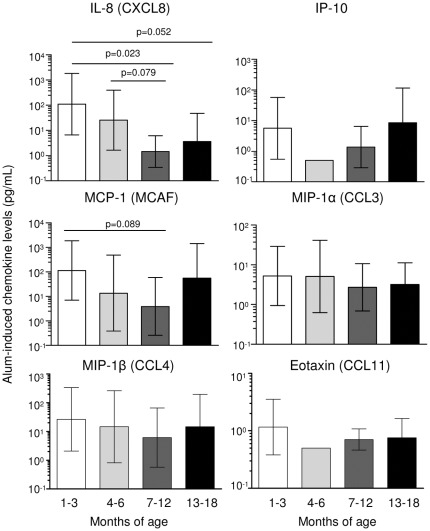
Alum-induced chemokine production with increasing age in infancy. Whole blood samples from PNG infants aged 1–3 months (n = 10, *white bars*), 4–6 months (n = 9, *light grey bars*), 7–12 months (n = 10, *dark grey bars*) or 13–18 months (n = 9, *black bars*) were stimulated with Alum. Presented are the geometric means and 95% confidence intervals (pg/mL) for each age group for background-adjusted chemokine responses. Significance level is indicated where p<0.10.

To study the maturation of TLR immune responses, whole blood cultures were stimulated with optimal doses of *Staphylococcus aureus*-derived lipoteichoic acid (LTA, TLR2-ligand; 20 µg/mL; InvivoGen, San Diego, USA), polyinosinic-polycytidylic acid (PolyIC, TLR3-ligand; 50 µg/mL; Sigma, Saint Louis, USA), *Escherichia coli*-derived lipopolysaccharide (LPS, TLR4-ligand; 1 ng/mL; Alexis Biochemicals, San Diego, USA) in the presence or absence of recombinant human IFN-γ (10 ng/mL; BD Biosciences Pharmingen, San Diego, USA); or gardiquimod (TLR7/8-ligand;10 µg/mL; InvivoGen, San Diego, USA).

To study maturation of NLR immune responses, blood samples were stimulated with optimal doses of γ-D-Glu-mDAP (iE-DAP, NOD1-ligand; 5 µg/mL; InvivoGen, San Diego, USA) and muramyl dipeptide (MDP, NOD2-ligand; 10 µg/mL; InvivoGen, San Diego, USA. To observe age-dependent responses to NALP3 inflammasome activation, cells were stimulated with aluminium potassium sulfate (Alum; 10 µg/mL, InvivoGen, San Diego, USA) alone or in the presence of LPS (particularly for measurement of mature IL-1β production as it is thought that the action of alum requires an additional signal provided by TLR co-stimulation for the initial synthesis of pro-IL-1β [23], [24]).

Whole blood samples were also cultured without stimulation (negative control for LTA, polyIC, LPS, gardiquimod, iE-DAP, MDP and alum-stimulated cultures) or with IFN-γ alone (negative control for LPS + IFN-γ-stimulated cultures) to correct for background responses. For alum + LPS-stimulated cultures, the LPS-stimulated response was subtracted as background.

Cultures were incubated at 37°C for 24 hours using the Aerogen Compact Atmosphere Generation System (Oxoid) according to previously published methods [31] to create a 5% CO_2_ level, and supernatants harvested and stored at -80°C before transferring to Perth via dry shipper for further experiments.

### *In Vitro* Cord and Peripheral Blood Mononuclear Cell (CBMC/PBMC) Cultures with Alum

CBMC had been isolated from cord blood of study children at the time of enrolment at birth; and PBMC were isolated from venous blood samples obtained from unrelated, healthy laboratory volunteers in PNG (n = 5). CBMC and PBMC were cryopreserved, transferred via dry shipper and stored in Perth (as previously described [32]) until use in functional assays. Thawed cells (2.5x10^5^/mL) were cultured in RPMI/10% NHI-FCS in 96-well plates at 37°C/5% CO_2_ with or without alum (10 µg/mL) in the presence or absence of LPS (1 ng/mL). Supernatants were collected at 24 hours and stored at -20°C.

### Cytokine and Chemokine Protein Detection

Cytokine levels were measured in supernatants using an in-house multiplex assay as previously described [32].

For Alum experiments, levels of the chemokines IL-8 (CXCL8), MCP-1 (MCAF), MIP-1α, MIP-1β, IP-10 and eotaxin in supernatants were measured using a Bioplex Pro Chemokine Assay kit (Human Group I: 6-plex panel, BIO-RAD, USA) according to the manufacturer’s instructions.

All samples were read on a Bio-Plex Suspension Array System (BIO-RAD, USA). Samples with concentrations below the detection limit were given the value corresponding to half the lowest concentration (3 pg/mL) that could be detected in this set of samples. The proportion of PRR-stimulated samples with cytokine concentrations below the detectable limit are shown in Table S1. Samples with concentrations above the level of detection i.e. 30,000 pg/mL (generally only IL-6) were further diluted and re-tested.

### Statistical Methods

As log-transformed cytokine responses were not normally distributed (assessed by QQ plots), differences in continuous variables were tested for significance using the Mann Whitney U test. To investigate the trajectory (slope) of the log-transformed cytokine response with increasing age, non-parametric Spearman rho correlations were performed and associated p-values used to assess whether the slope was significantly different from zero. Positive and negative coefficients suggest respective increases or decreases in cytokine responses across ordered age levels. Differences were considered to be statistically significant when p-values were less than 0.05. All analyses were performed using the statistical package SPSS 15.0.

## Results

### Infant Characteristics

Characteristics of the infant study population at the time of follow-up are described in Table 1. All children were afebrile at the time of assessment. Fifteen percent (7/46) of the children were hospitalized at least once prior to follow-up, mainly for upper and lower respiratory tract infections (data not shown).

### Maturation of TLR and NLR Immune Responses During Infancy

PNG infants aged 1–3, 4–6, 7–12 and 13–18 months old were compared for immune responses to a range of innate stimuli including TLR and NLR agonists (Figure 1).

A progressive and significant age-related increase in TLR2 and TLR3-mediated inflammatory cytokine production, including IL-6, IL-1β and IFN-γ was observed (Figure 1 and Table 2). A similar increase was observed for TLR3-induced IL-10 responses (Table 3). TLR7/8-induced responses were found to be similar across age groups (Figure 1 and Tables 2, 3, and 4). There were no clear age-related changes in TNF-α production, except for a significant decline in TLR4-induced levels between 1–3 and 7–12 months of age (Table 4). The innate production of IL-12 generally remained low and unchanged throughout infancy, except in response to TLR3 stimulation and TLR4/IFN-γ-primed stimulation, whereby trends for increasing IL-12 with age were observed (Figure 2).

With respect to NOD-associated pathways, infant responses remained stable between 1 and 18 months of age (Figure 1, Tables 2, 3, and 4). In contrast to TLR-induced IL-1β responses, NALP3 inflammasome (alum)-induced IL-1β production significantly declined with increasing age (when TLR co-stimulation was provided) (Table 2 and Figure 1).

### Alum Responses

We further examined alum-induced immune responses. Firstly, alum-induced IL-1β responses were compared between CBMC and PBMC of unrelated adult PNG donors and found to be significantly higher in CBMC (Figure 3). Together with the observation of decreasing alum-induced IL-1β levels during the first 18 months of life, these findings imply that in this population the alum response is likely highest at birth and progressively declines throughout infancy and childhood. Secondly, the production of additional alum-induced chemokines was assessed: CXCL8 (IL-8) and MCP-1 (MCAF) also tended to decline with increasing age in infancy, including a significant decline in CXCL8 during the first 12 months of life (Figure 4). There were no detectable age-related changes in any other alum-induced chemokines measured including IP-10, MIP-1α (CCL3), MIP-1β (CCL4) and eotaxin (CCL11) (Figure 4).

## Discussion

In this study we have described postnatal innate immune responses in relation to age in infants born in Papua New Guinea. To our knowledge, this is the first study to address age-related responses to NLR and inflammasome activation during infancy in any population.

The most important finding was an age-related decline in inflammatory responses to alum including IL-1β and CXCL8 (IL-8), in particular during the first 12 months of life. This observation could be of relevance for vaccine design and the implementation of immunization schedules. Alum in the form of aluminum hydroxide, aluminum phosphate, aluminum hydroxyphosphate sulfate, or the here-studied aluminum potassium sulfate, is used as an adjuvant in many pediatric vaccine formulations aiming to enhance immune responses to vaccine antigens [33], [34]. Following vaccination, the alum-induced activation of the NALP3 inflammasome results in the induction of Th2-associated inflammation and B cell isotype switching [25], [35]. Moreover, alum induces chemokines such as CXCL8 that recruit inflammatory cells to the site of vaccination and likely further boost vaccine responsiveness [25], [36]. Our finding that the immuno-stimulatory effects of alum change with increasing age in infancy could therefore potentially point at differences in the quality and/or quantity of the protective immune response generated by alum-containing vaccines at various stages of life. While the reason(s) for these age-related differences are unclear to us, one possibility is that tolerance to alum may occur following repeated exposure to alum-containing vaccines at birth and throughout infancy. Interestingly, in mice, effector and memory CD4^+^ T cells have been shown to selectively block NALP3 inflammasome activation in an antigen-specific manner to prevent potentially harmful inflammation [37]. It has been speculated that such CD4^+^ memory cells generated during primary immunisation dampen the NALP3-associated effects of alum upon subsequent exposure to booster vaccinations [38]. While this has not yet been demonstrated in humans, it may be a mechanism by which alum-induced inflammatory responses progressively decline during infancy. Further investigations are needed to determine whether these age-dependent changes in the inflammatory response to alum alter vaccine efficacy and whether this response pattern is unique to PNG infants or can be replicated in other human infant populations.

A second finding of this study was that innate IL-10 responses either increased or remained stable with age in the PNG study infants: for example, IL-10 responses to TLR3 increased with age, whilst responses to other studied TLR and NLR ligands remained constant. This is in contrast to observations for children living in high-income settings for whom TLR-mediated IL-10 responses have been reported to decline between birth and 2 years of age [12]; however, is in accord with findings for Gambian infants for whom IL-10 responses to TLR ligands were reported to remain stable to 12 months of age [10]. In line with the hygiene hypothesis, this may suggest population differences in anti-inflammatory responses throughout infancy. Speculatively, the persistence of high IL-10 responses might protect against the development of tissue damaging inflammation following frequent immune challenge; but on the other hand, it may also contribute to increased susceptibility to infections and may alter vaccine responses in these populations [27].

Thirdly, in line with other studies including those performed in high-income settings, pro-inflammatory responses to TLR activation, particularly to TLR2 and TLR3 agonists, increased with age in our PNG infant study population. This included increased IL-6, IL-1β and IFN-γ, as well as increased TLR3-induced IL-12 production. These findings suggest that TLR2 and TLR3 pathways are subject to universal intrinsic ‘immune maturation’. No clear changes in TNF-α production levels were observed across age groups in our study, except for an age-related decrease in response to TLR4 activation. Findings on TNF-α responses throughout infancy are conflicting: studies from Europe, North America and Australia have reported increasing LPS-induced TNF-α levels over the first year of life [11]–[13], whereas in Gambian infants, LPS-induced TNF-α responses remained stable between 1 and 12 months of age [10]. Thus, there may be population differences in age-related patterns of TNF-α production during infancy; which we speculate is potentially a result of differing environmental conditions. In line with the observed increasing innate IL-10 responses, down-regulation of TNF-α during infancy may further protect against harmful inflammatory responses under the high microbial burden of the PNG setting. On an additional note, TLR7/8-induced TNF-α production was not particularly high in our study population, in contrast to what has been reported during early life in other populations [10], [39]; at this stage it is unclear whether agonist choice (gardiquimod versus R-848, imidazoquinoline congener 3 M-003 or thiazoloquinoline CLO75) and/or inherent population differences explain these inconsistencies.

There is increasing evidence that the trajectory of early immune development may affect the risk of developing diseases such as allergy. Tulic and colleagues recently demonstrated that inflammatory (IL-1β, TNF-α, IL-6 and IL-12) responses to TLR activation were low at birth but increased with age in non-allergic Australian children, while the opposite trends were reported for allergic children [28]. In line with findings for Australian non-allergic children, we found that TLR-induced inflammatory (IL-1β, IL-6, IFN-γ and IL-12) responses in PNG children progressively increased during infancy. While we have not determined the allergic status of the children in our study, another study performed in a comparable population in PNG found no evidence of allergic sensitization in any of the individuals tested [40]. Together with our other observations of quiescent neonatal antigen presenting cell (APC) function in PNG newborns and higher innate immune responsiveness in Australian newborns (Lisciandro JG, manuscript in preparation), this suggests that protection from allergic disease is characterized by low innate immune responsiveness in early life, followed by progressive postnatal maturation of inflammatory responses. Notably, prenatal environmental exposures that influence the neonatal innate immune system [41], [42] likely play a significant role in determining the trajectory of postnatal immune development and subsequent risk for allergic disease.

There were a number of limitations to this exploratory study. Firstly, the sample size was relatively small. Secondly, in accordance with findings by Blimkie and colleagues [43], we were not able to make direct comparisons between cord and infant responses since cord innate responses were earlier measured using *in vitro* CBMC stimulations, while infant responses were measured in whole blood cultures, owing to the low volume of venous blood obtainable from infants. Despite these limitations, this study has demonstrated important changes in the ontogeny of innate immune pathways during infancy that appear to differ from findings for developed world populations. Larger-sized confirmatory studies that include birth as a time-point will provide further valuable insight into the ontogeny of innate immune pathways in resource-poor settings.

In conclusion, the ontogeny of innate immune responses may vary between geographically diverse populations. Age-related changes and geographical diversity in innate immune function may explain differences between and within populations in susceptibility to both infectious and non-communicable diseases, and have important implications for vaccine design and implementation of vaccine schedules. Further studies particularly addressing the ontogeny of responses to existing and candidate vaccine adjuvants that are aimed at enhancing innate immune function in early infancy require further attention.

## Supporting Information

Table S1 **Proportions of non-responders in the infant population.**
(DOC)
