# Supplementary material for: Ontogeny of Toll-Like and NOD-Like Receptor-Mediated Innate Immune Responses in Papua New Guinean Infants
Source: PLoS One. 2012 May 23;7(5):e36793. doi: 10.1371/journal.pone.0036793 (PMC3359332; doi:10.1371/journal.pone.0036793)
Supplement: Table S1 — Proportions of non-responders in the infant population. (DOC) [file pone.0036793.s001.doc]

**SUPPORTING INFORMATION**

**Ontogeny of Toll-like and NOD-like Receptor-mediated Innate Immune Responses in Papua New Guinean Infants**

Joanne G. Lisciandro (BSc/Hon)^1,2^, Susan L.Prescott (MD, PhD)^2^, Marie G. Nadal-Sims (BSc/Hon)^1^, Catherine J. Devitt (BSc/Hon)^1^, William Pomat (MSc)^3^, Peter M. Siba (PhD)^3^, Meri C. Tulic (PhD)^2^, Patrick G. Holt (DSc, FAA) ^1^, Deborah Strickland (PhD)^1^, Anita H.J. van den Biggelaar (PhD)1^

1. Telethon Institute for Child Health Research, Centre for Child Health Research, University of Western Australia, Perth, Australia
2. School of Paediatrics and Child Health, University of Western Australia, Perth, Australia
3. Papua New Guinea Institute of Medical Research, Goroka, Papua New Guinea

^ Current affiliation: Crucell, Archimedesweg 4-6, 2333 CN, The Netherlands

**Corresponding author**: Joanne G. Lisciandro, Telethon Institute for Child Health Research, Centre for Child Health Research, University of Western Australia, Perth, Australia. Phone: +61-8-94897764, Email: joannel@ichr.uwa.edu.au

**SUPPLEMENTARY TABLE**

**Table S1. Proportions of non-responders in the infant population**

|  | **IL-10** | **IL-6** | **TNF-α** | **IL-1β** | **IL-12** | **IFN-γ** |
| --- | --- | --- | --- | --- | --- | --- |
| **TLR2** | 2/67 (3%) | 2/67 (3%) | 5/67 (7%) | 2/67 (3%) | 24/67 (36%) | 12/67 (18%) |
| **TLR3** | 1/67 (1%) | 2/67 (3%) | 1/67 (1%) | 1/67 (1%) | 11/67 (16%) | 7/67 (10%) |
| **TLR4** | 1/67 (1%) | 0/67 (0%) | 4/67 (6%) | 1/67 (1%) | 15/67 (22%) | 7/67 (10%) |
| **TLR4†** | 4/67 (6%) | 3/67 (4%) | 7/67 (10%) | 5/67 (7%) | 10/67 (15%) | 14/67 (21%) |
| **TLR7/8** | 3/67 (4%) | 2/67 (3%) | 6/67 (9%) | 3/67 (4%) | 20/67 (29%) | 9/67 (13%) |
| **NOD1** | 50/67 (75%) | 32/67 (48%) | 46/67 (69%) | 29/67 (43%) | 43/67 (64%) | 45/67 (67%) |
| **NOD2** | 23/67 (34%) | 9/67 (13%) | 30/67 (45%) | 17/67 (25%) | 39/67 (58%) | 40/67 (60%) |
| **NALP3** | 59/67 (88%) | 43/67 (64%) | 46/67 (69%) | 40/67 (60%) | 41/67 (61%) | 39/67 (58%) |
| **NALP3♦** | 25/67 (37%) | 31/67 (46%) | 35/67 (52%) | 29/67 (43%) | 42/67 (63%) | 40/67 (60%) |

Data show the proportion of samples that did not respond to PRR stimulation (i.e. concentration of cytokine was below the detectable limit of 3 pg/mL).

♦ denotes LPS co-stimulation used

**†** denotes IFN-γ priming used
